# Proceedings of the Border Medical Society. Instituted June 27, 1838

**Published:** 1840-04

**Authors:** 


Art. XII.—*Proceedings of the Border Medical Society. Instituted June* 27, 1838. Parts I. and II.—*Kelso,* 1838-9. 8vo, pp. 26.

We have been much gratified by the perusal of these small fasciculi, and still more by contemplating the honorable and zealous spirit from which they spring. To see established an association for the cultivation of medical science and the promotion of the noblest and most dignified objects of our profession, in so limited and retired a district as that to which this society is confined, and, yet more, to find this small body of country practitioners (not exceeding twenty-four in all) reading at their meetings, and then printing for circulation among the members and their friends, excellent medical memoirs, is surely to witness signs of times which our forefathers could have hardly dreamed of, and of which we may be justly proud. The following are the praiseworthy objects of this society:

"1. The increase of knowledge of the medical topography of the district, through statistical, meteorological, geological, and botanical enquiries. 2. Collection of useful information, whether speculative or practical, through original essays or reports, founded on private practice, on that of public institutions, or on medico-legal investigations before the provincial courts. 3. The investigation of epidemic and endemic diseases; tracing their connexion with peculiarities of soil or climate, or with the circumstances, habits, and occupations of the people; and ascertaining, in as far as the means of knowledge will permit, such local causes as may influence the character of diseases occurring within the district. 4. Maintenance of the honour and respectability of the profession generally in the district; and promotion of friendly intercourse and free communication amongst its members."

In another part of our Journal we shall select one or two passages from the "Proceedings" of this Society, to which we cordially wish all success. We recommend the following excellent observation, which forms the concluding paragraph of a well-written report on the diseases of Kelso, by Dr. Wilson, to the attention of all those (and we fear they are not few) who are disposed to undervalue the case-keeping and note-taking industry of practitioners in the ordinary walks of the profession:

"It is, of course, manifest, that we are not to expect important discoveries to start up before us at every stage of our progress; neither is the detection of novelties to be considered as by any means essential to our full success. He who gives to the most familiar truth a greater stability or a more extended application, or who confirms, by additional proofs, principles which may have been previously discovered but have never been fully evinced, and so contributes to render more secure the base upon which science is founded, is engaged in no unimportant or ungrateful task. It is by the spirit of cautious observation and induction, by the slow stealing from fact to fact, that the empire of knowledge is to be firmly established, if its first conquests are sometimes to be achieved by more brilliant and rapid movements. And surely, engaged as we are in the study of one of the noblest and most comprehensive departments of human science, there is not one of us so mean that he is entitled to derive no incitement to perseverance from the words of the illustrious Bacon:—"Non abs re fuerit etiam notare, facultates vel mediocres, si in magnos viros aut res magnas inciderint, graves et insignes interdum producere effectus."

